# Correlation between liver stiffness measurement and hepatocellular carcinoma risk: A case control study

**DOI:** 10.6026/973206300220169

**Published:** 2026-01-31

**Authors:** Abhinav Garg, Yogesh Kumar Gupta, Sanjeev Puri, Staphy Garg, Varun Mehta, Dinesh Gupta

**Affiliations:** 1 Consultant, Vardhman Mahavir Hospital, Patiala, Punjab, India; 2 Department of Gastroenterology, Dayanand Medical College and Hospital, Ludhiana, Punjab, India; 3 Department of Otolaryngology, Dayanand Medical College and hospital, Ludhiana, Punjab, India; 4 Department of Pulmonary Medicine, Government Medical College, Patiala, India; 5 Department of Gastroenterology, Dayanand Medical college and Hospital Ludhiana, Punjab, India; 6 Consultant, Fortis Hospital, Ludhiana, Punjab, India

**Keywords:** Hepatocellular carcinoma, liver stiffness measurement, transient elastography, cirrhosis, FibroScan, non-invasive diagnostics

## Abstract

Liver stiffness measurement (LSM) using transient elastography (TE) poorly predicts hepatocellular carcinoma risk in cirrhotic patients, despite its established role in fibrosis assessment. This case-control study compared 30 hepatocellular carcinoma (HCC) patients with cirrhosis to 30 cirrhosis-only patients using FibroScan® TE for LSM. HCC patients were older, mostly male, with Hepatitis C virus (HCV) as the primary cirrhosis cause, followed by alcohol; median LSM was higher in HCC (58.0 kPa) versus non-HCC (24.5 kPa). Stratum-specific likelihood ratios rose with LSM and multivariate analysis confirmed age, HCV, alcohol and LSM as independent HCC predictors; LSM ≥39.45 kPa showed 83% sensitivity and 80% specificity for HCC detection in cirrhosis. Elevated LSM strongly associates with HCC risk, supporting TE as a non-invasive tool, especially with HCV/alcohol factors.

## Background:

Hepatocellular carcinoma (HCC) represents a major global health burden, ranking as the sixth most frequently diagnosed cancer worldwide and accounting for approximately 7% of all malignancies [1, 2]. More alarmingly, it stands as the fourth leading cause of cancer-related mortality [2], reflecting its aggressive nature and often late-stage diagnosis. The pathogenesis of HCC is multifactorial, with risk factors including viral hepatitis infections (HBV and HCV), chronic alcohol consumption, metabolic dysfunction-associated steatotic liver disease (MASLD) and various genetic and autoimmune conditions [1, 2]. However, the development of advanced liver fibrosis and subsequent cirrhosis remains the single most significant risk factor, with epidemiological studies demonstrating that 80-90% of HCC cases arise in cirrhotic livers [3]. For decades, liver biopsy has served as the gold standard for assessing hepatic fibrosis and steatosis [4]. However, this invasive procedure suffers from several well-documented limitations. Its diagnostic accuracy is constrained by sampling variability (evaluating only about 1/50,000 of the total liver volume), inter-observer variability and a sensitivity below 80% [4, 5-6]. Furthermore, liver biopsy carries non-negligible risks of complications including pain, hemorrhage (occurring in 0.3-0.5% of cases) [7] and rarely life-threatening events such as hemoperitoneum or inadvertent organ puncture. These limitations, combined with the substantial costs associated with the procedure (including hospitalization, specialist fees and pathological analysis), have driven the search for reliable non-invasive alternatives. Transient elastography (TE) has emerged as a promising non-invasive modality for liver fibrosis assessment [7, 8]. This innovative technology measures liver stiffness by generating low-frequency (50Hz) shear waves through an ultrasound transducer and calculating their propagation velocity through hepatic tissue [9]. The principle is based on the fact that stiffer fibrotic tissue transmits these waves faster than normal parenchyma. TE offers several advantages over traditional biopsy: it evaluates a liver volume approximately 100 times larger (a cylindrical section of 1cm diameter x 4cm length), provides immediate quantitative results expressed in kilopascals (kPa) and has excellent reproducibility when proper quality criteria are met (≥10 valid measurements, success rate >60% and IQR/M ratio ≤0.3) [7]. Recent research has established strong correlations between elevated liver stiffness measurements (LSM) and both the severity of liver fibrosis and the risk of hepatic complications, including portal hypertension and HCC [10-11]. These findings suggest that TE may serve not only as a diagnostic tool for fibrosis staging but also as a prognostic indicator for HCC development in high-risk patients. Therefore, it is of interest to evaluate the relationship between TE-derived liver stiffness measurements and HCC occurrence in cirrhotic patients, with the ultimate goal of refining HCC surveillance strategies and improving early detection rates in this vulnerable population.

## Materials and Methods:

## Study design and population:

This case-control study was conducted at the Department of Medicine and Gastroenterology, Dayanand Medical College and Hospital, Ludhiana, between October 2019 and March 2021. We enrolled 60 patients with cirrhosis, comprising two matched groups: Group A (n=30) with concurrent hepatocellular carcinoma (HCC) and Group B (n=30) without HCC.

## Inclusion criteria:

For Group A (cirrhosis with HCC):

[1] Confirmed cirrhosis of any etiology (compensated or decompensated)

[2] HCC diagnosis meeting EASL criteria (either non-invasive imaging characteristics or histopathological confirmation)

[3] Age >18 years

For Group B (cirrhosis without HCC):

[1] Cirrhosis of any etiology (compensated only)

[2] Absence of HCC confirmed by negative ultrasonography and triphasic CT/MRI when indicated

## Exclusion criteria (both groups):

[1] BMI >30 kg/m^2^

[2] ALT levels >10x upper limit of normal

[3] Concurrent heart failure

[4] Local skin lesions at FibroScan probe site

[5] Pregnancy or active implantable medical devices

## Clinical evaluation:

All participants underwent comprehensive assessment including:

[1] Detailed medical history and physical examination

[2] Evaluation for cirrhosis complications (ascites, jaundice, variceal bleeding, encephalopathy)

[3] BMI calculation

[4] Systematic respiratory, cardiovascular and gastrointestinal examinations

## Etiological workup:

Chronic liver disease etiology was established using standard criteria:

[1] HBV: Positive HBsAg serology

[2] HCV: Positive anti-HCV antibodies

[3] Alcohol-related: Daily consumption ≥40g for ≥5 years

[4] Other etiologies diagnosed per current guidelines

## Liver stiffness measurement:

Transient elastography was performed using FibroScan® (EchoSens) by a dedicated operator blinded to clinical data. The procedure involved:

[1] Right lobe measurements through intercostal spaces

[2] Patient positioning in dorsal decubitus with right arm abducted

[3] Acquisition of ≥10 valid measurements

[4] Maintenance of measurement depth between 25-65mm

[5] Quality criteria: success rate >60%, IQR/M ≤0.3

## Statistical analysis:

Descriptive statistics (mean±SD, frequencies, percentages) Normality assessment using Kolmogorov-Smirnov test Group comparisons with Student's t-test (parametric) or Mann-Whitney U test (non-parametric), Categorical variable analysis with χ^2^ or Fisher's exact tests. ROC curve analysis for diagnostic accuracy. Multivariate logistic regression for risk factor identification. All analyses performed using SPSS v21 (IBM Corp.), with p<0.05 considered significant.

## Results:

Maximum patients in Group A were from age group of 61-70 years (46.7%) whereas maximum patients in Group B were from age group of 51-60 years (40%). The mean age in Group A was 60.10 years ± 8.14, whereas mean age of Group B was 55.23 years ± 8.50(60.10 years ± 8.14 v/s 55.23 years ± 8.50). Patients with HCC were elderly individuals. In Group A 90.0 % of patients were Male, 10.0% were female In Group B 93.3 % of patients were Male, 6.7% were female (Table 1). In Group A 33.4% of patients had alcohol as etiology, 50.0% of patients had HCV as etiology, 6.7% of patients had HBV as etiology, 6.7% of patients had NASH as etiology and 3.33% had coexisting HCV and HBV as etiology. In Group B 40.0% of patients had alcohol as etiology, 20.0% of patients had HCV as etiology, 6.7% of patients had HBV as etiology and 33.33% of patients had NASH as etiology (Table 2). In our study Group B had compensated liver disease, whereas in Group A had 6 patients (20%) with ascites, 8 patients (26.7%) had jaundice, 2 patients (6.7%) had variceal bleed and 2 patients (6.7%) had hepatic encephalopathy. In our study 31 patients (51.7%) had space occupying lesion as evident Triphasic CT out of which 30 had malignant lesions LIRADS V and 1 patient had Benign lesion. 12 patients (20.0%) in our study had portal vein thrombosis as evident by CT scan. Out of which 11(36.7%) were having HCC and 1(3.3%) was having cirrhosis with HCC (Table 3). In our study we took cases and controls with similar characteristics. The mean BMI in Group A was 25.89± 2.19 whereas in Group B the mean BMI was 26.68±2.88. Patients in Group A more platelet count 147.435 x 103± 97.141 x 103as compared to Group B patients where mean platelet count was 130.933 x 103± 56.836 x 103. Patients with HCC had mean serum bilirubin of 1.34 ±0.74 mg/dl where as those without HCC had 0.99 ± 0.49 mg/dl both the groups had SGOT and SGPT below 5 times of the upper limit. As expected the serum AFP levels were more in those with HCC, the mean serum AFP level in Group A was 2257.35 ± 6378.69 and in Group B it was 7.28 ± 10.80 (Table 4). In our study the median Controlled attenuation parameter value of Group A patients was 259.00 with IQR of 233-286 and 261.50 with IQR of 206-293 in Group B patients. The median Liver stiffness measurement of Group A patients was 58.00 with IQR of 46-70 and 24.50 with IQR of 18-37 in Group B patients (Table 5). The likelihood ratio increased as we moved up the stratum. The likelihood ratio for group < 30 is 0.2, Likelihood ratio for group 30-50 is 1 and group>50 kPa is 4.2. As we increased above the stratum the likelihood ratio for HCC increased. The likelihood ratio for HCC in patients with cirrhosis due to HCV increased as we moved up the stratum. The likelihood ratio for group < 30 is 0.15, Likelihood ratio for group 30-50 is and group >50 kPa is 4.8. As we increased above the stratum the likelihood ratio for HCC increased. The likelihood ratio for HCC in patients with cirrhosis due to alcohol increased as we moved up the stratum. The likelihood ratio for group < 30 is 0.34, Likelihood ratio for group 30-50 is 0.8 and group >50 kPa is 3.6. As we increased above the stratum the likelihood ratio for HCC increased (Table 6). In a univariate analysis age, LSM, Etiology of cirrhosis (hepatitis C and alcohol), diabetes mellitus, total bilirubin were risk factors associated with HCC, whereas sex, platelet count, albumin and AFP were not associated as risk factors for HCC (Table 7). In a multivariate analysis age, LSM, Etiology of cirrhosis (hepatitis C and alcohol) were independent risk factors associated with HCC, whereas platelet count, bilirubin, albumin and AFP were not associated as risk factor for HCC (Table 8).

## Discussion:

Liver fibrosis has been reported to be correlated with the risk of HCC and currently liver biopsy is considered as the best standard of care for the assessment of liver fibrosis. In this study, the mean age of cirrhosis patients with HCC (60.1 years) was higher than those without HCC (55.53 years), consistent with findings from Nahon *et al.* (65.7 vs. 57.1 years), Masuzaki *et al.* (67.5 vs. 70.8 years) and Kuo *et al.* (61.8 vs. 51 years) [12, 13-14]. Male predominance was observed in both groups (90% with HCC, 93.3% without HCC), aligning with global trends and prior studies showing higher HCC incidence in males (Nahon *et al.*: 72.7% with HCC, 63.3% without; Kuo *et al.*: 61.7% with HCC, 84% without) [12, 14]. These findings reinforce the association between older age, male sex and HCC risk in cirrhotic patients. It has been seen throughout the world that males have higher incidence rate as compared to females [15]. In this study, HCV was the most common etiology in cirrhosis with HCC (50%), followed by alcohol (33.4%), while in cirrhosis without HCC, alcohol (40%) and NASH (33.3%) predominated. These findings contrast with Nahon *et al.* [12] (HCV ~65% in both groups) and Kuo *et al.* [14] (HBV-dominant HCC, HCV-dominant non-HCC), highlighting regional variations in underlying causes. The higher alcohol-related cirrhosis in non-HCC patients suggests its role in progression before malignant transformation. Performing FibroScan in patients with ascites can be challenging. In our study, Group B patients had compensated liver disease, while Group A included six patients with ascites-mostly minimal and non-tapable. In cases where ascites was present, therapeutic paracentesis was performed before FibroScan. Similar to findings by Kohlhaas *et al.* [16], liver stiffness measurements were not significantly affected by ascites unless hepatic congestion was present. Another limitation is jaundice; in our study, nine patients had elevated bilirubin due to decompensated liver disease and FibroScan was performed only when levels were below 2.5 mg/dL. The mean bilirubin in HCC patients was 1.34 ± 0.74, compared to 0.99 ± 0.49 in non-HCC patients, aligning with Kuo *et al.*'s findings (1.63 ± 4.39 vs. 1.02 ± 0.88) [14].

In our study, triphasic CT revealed space-occupying lesions (SOLs) in 32 patients, with 30 classified as LI-RADS V (highly suggestive of HCC) and only two as benign lesions. The remaining 28 patients had no SOLs. Multiple cohort studies have demonstrated that increased liver stiffness correlates with a higher risk of HCC. A study by Yoshida *et al.* [17] on 2800 chronic hepatitis C patients identified liver fibrosis as the strongest risk factor for HCC, followed by age and male sex. FibroScan quantifies liver stiffness as a continuous variable (LSM), offering an advantage over liver biopsy. In our cohort, HCC patients across all etiologies (HCV, HBV, alcohol, NASH) exhibited higher LSM values than non-HCC patients. Univariate analysis confirmed that elevated LSM, along with advanced age and other biological factors, was strongly associated with HCC. These findings align with the study by Nahon *et al.* where increased LSM and older age were significant predictors of HCC development [12]. Our study identified LSM and age as independent predictors of HCC, consistent with prior research [12, 13, 18]. Unlike other studies, AFP did not show significant association, supporting its limited reliability in HCC screening [19]. Elevated ALT was excluded to optimize LSM accuracy, potentially introducing selection bias but strengthening result validity [20, 21]. These findings highlight LSM's utility in HCC risk stratification, particularly alongside age, while emphasizing the variable relevance of traditional biomarkers across etiologies. In our study, HCV-related HCC patients exhibited higher median LSM values than alcohol-related cases, suggesting HCV contributes more significantly to liver stiffness- contrasting with Nahon *et al.*'s findings where alcohol-associated cirrhosis showed higher stiffness [12]. Stratifying patients by LSM (<30, 30-50, >50 kPa), we found HCC likelihood ratios increased sharply with higher stiffness (LR 0.2, 1.0 and 4.2, respectively), aligning with Kuo *et al.*'s data [14]. This highlights LSM's role in HCC risk stratification, particularly in HCV, though further studies are needed for validation. Our analysis revealed that HCC likelihood ratios (LR) increased with higher LSM strata across etiologies. In HCV-related cirrhosis, LR rose sharply from 0.15 (<30 kPa) to 4.8 (>50 kPa), mirroring findings by Masuzaki *et al.* [13] and Adler *et al.* [3]. Alcohol-related cirrhosis showed a similar trend (LR: 0.34 to 3.6), though the risk escalation was less pronounced than in HCV. While HBV and NASH subgroups could not be stratified due to sample size limitations, the consistent pattern underscores LSM's universal predictive value for HCC risk stratification, with HCV demonstrating the strongest association.

## Conclusion:

A wide range of LSM is observed according to the cause of the underlying liver disease and the presence of HCC is associated with higher values in these patients in patients with cirrhosis without HCC. A cut-off 39.45 kPa is useful with a sensitivity of 83% and a specificity of 80% for predicting the presence of HCC.

## Figures and Tables

**Table 1:** Age and sex distribution of subjects in Group A and Group B

		**GROUP A**		**GROUP B**		**Total**
		**No. of cases**	**%age**	**No. of cases**	**%age**	
AGE	<50	3	10.00%	11	36.70%	14
	51-60	10	33.30%	12	40.00%	22
	61-70	14	46.70%	6	20.00%	20
	>70	3	10.00%	1	3.30%	4
SEX	F	3	10.00%	2	6.70%	5
	M	27	90.00%	28	93.30%	55
	Total	30	100%	30	100%	60

**Table 2:** Etiology of CLD in Group A and Group B

	**GROUP A**		**GROUP B**	
	**No. of cases**	**%age**	**No. of cases**	**%age**
HEPATITIS B	2	6.70%	2	6.70%
HEPATITIS C	15	50.00%	6	20.00%
ALCOHOL	10	33.40%	12	40.00%
NASH	2	6.70%	10	33.30%
HBV and HCV	1	3.33%	-	-
TOTAL	30	100%	30	100%

**Table 3:** Liver compensations status of patients with Cirrhosis and Triphasic CT scan findings in Group A and Group B

		**GROUP A**		**GROUP B**	
		**No. of cases**	**%age**	**No. of cases**	**%age**
Liver Disease Compensated	Absent	11	36.70%	0	0.00%
	Present	19	63.30%	30	100.00%
Ascites (Decompensated)	Absent	24	80.00%	30	100.00%
	Present	6	20.00%	0	0.00%
Jaundice Decompensated	Absent	22	73.30%	30	100.00%
	Present	8	26.70%	0	0.00%
Variceal Bleed Decompensated	Absent	28	93.30%	30	100.00%
	Present	2	6.70%	0	0.00%
Hepatic Encephalopathy Decompensated	Absent	28	93.30%	30	100.00%
	Present	2	6.70%	0	0.00%
Coagulation Defect Decompensated	NO	30	100.00%	30	100.00%
Triphasic CT scan					
Space occupying lesion	Absent	0	0.00%	28	93.34%
	Present	30	100.00%	2	6.64%
Portal vein thrombosis	Absent	19	63.30%	29	96.70%
	Present	11	36.70%	1	3.30%

**Table 4:** Baseline characteristics of patients in Group A and Group B

	**GROUP A**		**GROUP B**		**p-value**
	**Mean**	**SD**	**Mean**	**SD**	
BMI	25.89	2.19	26.68	2.88	0.241
Platelet	147435.47	97141.65	130933.33	56836.69	0.425
Bilirubin(T)	1.34	0.74	0.99	0.49	0.034
Bilirubin(D)	0.69	0.5	0.41	0.27	0.009
SGOT	89.47	80.22	68.9	48.55	0.235
SGPT	56.45	49.73	62.4	64.15	0.69
ALP	163.13	97.31	112.43	90.84	0.041
GGT	100.4	67.18	132.5	160.03	0.315
Albumin	3.18	0.61	3.16	0.67	0.887
AFP	2257.35	6378.69	7.28	10.8	0.058

**Table 5:** Relationship of LSM and CAP with disease in Group A and Group B(median value)

	**GROUP A**		**GROUP B**		**p-value**
	**Median**	**IQR**	**Median**	**IQR**	
Fibroscan Controlled Attenuation Parameter	259	233-286	261.5	206-293	0.477
Fibroscan Liver Stiffness	58	46-70	24.5	18-37	0

**Table 6:** Stratum-specific likelihood ratio analysis of liver stiffness, liver stiffness for HCV and liver stiffness for Alcohol

		**GROUP A**		**GROUP B**		**Total**	**SSLR(95% CI)**
		**No. of**	**%age**	**No. of**	**%age**		
		**cases**		**cases**			
	< 30	4	13.30%	20	66.70%	24	0.2 (0.08-0.52)
LSM	30-50	5	16.70%	5	16.70%	10	1 (0.32-3.10)
	> 50	21	70.00%	5	16.70%	26	4.2 (1.82-9.67)
Total		30	100.00%	30	100.00%	60	
	< 30	2	13.30%	5	100%	7	0.15(0.04-0.55)
LSM for HCV	30-50	1	6.67%	0	0%	1	-
	> 50	12	80.00%	1	0%	13	4.8(0.79-29)
Total		15	100.00%	6	100.00%	21	
LSM for alcohol	< 30	2	20.00%	7	58.33%	9	0.34(0.09-1.29)
	30-50	2	20.00%	3	36.00%	5	0.8(0.16-3.88)
	> 50	6	60.00%	2	16.67%	8	3.6(0.92-14.06)
Total		15	100.00%	12	100.00%	22	

**Table 7:** Univariate analysis of Bio clinical features associated with the risk of HCC

**Characteristics**	**P-value**
AGE	0.038
SEX	0.64
HEPATITIS C	0.033
ALCOHOL	0.032
PLATELET COUNT	0.425
DIABETES MELLITUS	0.015
BILIRUBIN(T)	0.034
ALBUMIN	0.887
AFP	0.058
LSM	<0.001

**Table 8:** Multivariate analysis of Bio clinical features associated with the risk of HCC

	**P-VALUE**	**Exp(B)**	**95% C.I. for EXP(B)**	
			**Lower**	**Upper**
AGE	0.01	1.168	1.038	1.313
PLATELET	0.668	1	1	1
COUNT				
BILIRUBIN(T)	0.231	2.344	0.581	9.457
ALBUMIN	0.539	0.681	0.2	2.315
AFP	0.268	1	1	1
LSM	0.011	1.052	1.012	1.094
HEPATITIS C	0.017	9.001	1.48	54.759
ALCOHOL	0.03	8.012	1.229	52.221
